# The role of filamentous matrix molecules in shaping the architecture and emergent properties of bacterial biofilms

**DOI:** 10.1042/BCJ20210301

**Published:** 2024-02-15

**Authors:** Jan Böhning, Abul K. Tarafder, Tanmay A.M. Bharat

**Affiliations:** Structural Studies Division, MRC Laboratory of Molecular Biology, Francis Crick Avenue, Cambridge CB2 0QH, U.K.

**Keywords:** bacteria, biofilms, extracellular matrix, microbiology, structural biology

## Abstract

Numerous bacteria naturally occur within spatially organised, multicellular communities called biofilms. Moreover, most bacterial infections proceed with biofilm formation, posing major challenges to human health. Within biofilms, bacterial cells are embedded in a primarily self-produced extracellular matrix, which is a defining feature of all biofilms. The biofilm matrix is a complex, viscous mixture primarily composed of polymeric substances such as polysaccharides, filamentous protein fibres, and extracellular DNA. The structured arrangement of the matrix bestows bacteria with beneficial emergent properties that are not displayed by planktonic cells, conferring protection against physical and chemical stresses, including antibiotic treatment. However, a lack of multi-scale information at the molecular level has prevented a better understanding of this matrix and its properties. Here, we review recent progress on the molecular characterisation of filamentous biofilm matrix components and their three-dimensional spatial organisation within biofilms.

## Introduction

Biofilms are multicellular ordered communities of bacteria that are the major mode of bacterial life found in nature [1–3]. Biofilms are organised, three-dimensional assemblies [4,5] that endow numerous survival advantages to constituent cells, including tolerance to antimicrobials, tolerance to extracellular physical stresses and protection from harmful external entities [6–9]. Biofilm formation begins when a planktonic cell attaches to an amenable surface [10], followed by growth and expansion into a colony, leading to biofilm maturation. During the maturation stage, bacterial cells secrete extracellular polymeric substances (EPS), including polysaccharides, filamentous proteins, extracellular DNA (eDNA) and other polymers forming an extracellular matrix (ECM) that surrounds bacterial cells and scaffolds the biofilm. The presence of an ECM is the defining characteristic and hallmark of all bacterial biofilms, and is responsible for many of the key properties exhibited by biofilms.

The biofilm matrix in many bacteria contains a wide-variety of polymeric components with spatial segregation and ordering [7]. Indeed, the three-dimensional architecture of the biofilm often includes pores, channels, and localised regions with differing physicochemical properties [11]. While different bacteria have differing molecular organisation within the ECM of biofilms, shared organisational principles have emerged from recent studies, because the components of the biofilm matrix must fulfil many defined functions. For instance, in the case of surface-attached biofilms, molecules in the ECM must provide adherence to the substrate. Furthermore, bacterial cells must adhere together to prevent unintended (and metabolically costly) disassembly of the biofilm due to random shear or mechanical stresses. Finally, EPS molecules within the ECM must protect bacterial cells from unwanted intrusion from predators (such as grazing protozoans), phages and chemical agents including antimicrobials. The protective function of the ECM is particularly relevant for biofilms formed during human infection [12], where biofilm formation can increase antibiotic tolerance of constituent bacteria by up to a 1000-fold [12].

Several recent molecular biology, cell biology and microscopy studies have improved our understanding of how the ECM is arranged in biofilms. Recent structural studies have linked atomic detail of constituent molecules with imaging data, permitting a multi-scale understanding of the emergent properties of biofilms. While numerous polymeric molecules have been implicated in sculpting the biofilm architecture (Table 1), in this review, we focus on components of the ECM of bacterial biofilms where multi-scale data is available, allowing us to infer the molecular basis of architecture and the emergent properties displayed by biofilms. Using these illustrative cases, we discuss how EPS molecules within the ECM endow special traits to the biofilm. Understanding the collective effect of the ECM on constituent cells as well as entire biofilms will illuminate the core principles that shape biofilms and their emergent properties.

**Table 1.** Examples of polymeric molecules implicated in shaping biofilm architecture

Molecule type	Name	Species	Structure and function	References
Proteins	Curli	*E. coli*, *Salmonella* spp., other Gram-negative bacteria	Functional amyloid with cross-β structure. Major proteinaceous component of biofilm ECM. Roles in structural maintenance of biofilm ECM, adhesion to surfaces, cell-cell adhesion, host cell adhesion and invasion.	[13–16]
	Fap	*P. aeruginosa*	Functional amyloid with predicted cross-β structure. Maintains structural integrity of biofilm ECM.	[17,18]
	PSM	*S. aureus*	Small, cytotoxic peptides that self-assemble into higher order amyloid-like fibres. PSMα3 and PSMβ2 fibres formed of α-helical peptides stacked laterally to form a cross-α fold. PSMα1 and PSMα4 assemble into canonical cross-β amyloid fibres. Role in structural scaffolding of biofilm formation.	[19–21,22]
	TasA	*B. subtilis*	Globular β-sandwich domain protein polymerising into fibres by donor-strand exchange of β-strand with neighbouring subunit. Major proteinaceous component of biofilm ECM required for structural integrity. Fibres show propensity to bundle.	[23,24]
	CupA-D	*P. aeruginosa*	Classical CUP pilus — putative rod-like structure. Fibres formed through donor-strand exchange of donor β-strand between neighbouring subunits. Role in initial adhesion to surfaces and microcolony formation.	[25–27]
	Csu	*A. baumannii*	Archaic CUP pilus — forms highly elastic fibres with a linear zigzag arrangement of subunits. Fibres formed through donor-strand exchange of donor β-strand between neighbouring subunits. Capped with a hydrophobic tip subunit mediating adhesion to abiotic surfaces. A major virulence factor with roles in maintaining biofilm structure and attachment to abiotic surfaces.	[28–31]
	CupE	*P. aeruginosa*	Archaic CUP pilus — forms fibres with a linear zigzag arrangement of subunits. Homologous to Csu. Capped with a hydrophobic tip subunit. Stabilises 3D architecture of mature biofilms.	[32,33]
	Type IV pili	Many species	Polymeric hydrophobic proteinaceous fibres composed of repeating pilin subunits often capped with an adhesin subunit. Implicated in early stages of biofilm formation. Twitching motility involved in microcolony formation.	[3,34,35]
	FimA	*E. coli*, *K. pneumoniae*, *S. marcescens*	Surface-attached type I Chaperone-Usher pilus. Forms 1–5 μm long fibres. Fibres formed through donor-strand exchange of β-strands between neighbouring subunits. Pilus capped by an adhesive subunit that binds mannosylated glycoproteins. Instrumental in biofilm formation and critical for initial attachment to abiotic surfaces using a ‘catch-bond’ mechanism.	[36–39]
	ECP (*E. coli* common pili)	*E. coli*	Composed of multiple copies of 18 kDa EcpA subunit. Fibres formed through donor-strand exchange of an N-terminal β-strand between neighbouring subunits. Dual role in biofilm formation and host cell recognition. Fibres show tendency to aggregate into well-ordered bundles postulated to be important for biofilm formation.	[36,40,41]
	Tad (tight adherence pili)	*P. aeruginosa*, *C. crescentus*, *A. actinomycetemcomitans*	Subset of type IVb pili expressed in both Gram-negative and positive bacteria. Promote surface colonisation, cell-to-cell attachment and biofilm cohesion.	[36,42]
	TCP (Toxin co-regulated pilus)	*V. cholerae*	Receptor for CTXϕ inovirus. Deletion of TCP disrupts microcolony formation and results in unstructured biofilms.	[36,43]
	Pf4	*P. aeruginosa*	Filamentous (pro)phage integrated into the *P. aeruginosa* genome — expression highly up-regulated on switch to biofilm lifestyle. Linked to an increase in *P. aeruginosa* pathogenicity. Capsid composed of interdigitated array of α-helical coat protein subunits surrounding a ssDNA genome. Pf4 phage filaments phase-separate into liquid crystalline structures termed tactoids. Increased antibiotic tolerance due to Pf4 tactoids encapsulating cells forming a diffusion barrier. Contributes to biofilm stability and resistance to desiccation. Acts as an immune decoy inhibiting clearance of *P. aeruginosa*.	[44–48]
	Bap	*S. aureus*	Cell surface-attached high molecular mass multi-domain proteins. N-terminal dumbbell-shaped domain is released after proteolytic cleavage and forms insoluble amyloid-like fibres through liquid-liquid phase separation in response to pH and Ca^2+^. Link environmental stimuli to formation of the biofilm ECM.	[49,50]
	Ag43, AIDA, tibA	*E. coli*	Members of the self-associating autotransporter (SAAT) family of proteins. Outer membrane anchored proteins that are independently transported across the membrane (type V secretion). Composed of a surface localised passenger domain and a C-terminal translocation domain which forms a β-barrel pore in outer membrane to transport passenger domain. Mediate bacterial aggregation by close range homotypic/heterotypic inter-cell association between SAATs. Ag43 promotes biofilm formation on abiotic surfaces. Autotransporters are widespread throughout Gram-negative bacteria with roles in host cell invasion and bacterial aggregation, for example Pertactin in *B. pertussis* and Hap in *H. influenzae*.	[51–53]
	P1 adhesin (Ag I/II)	*S. mutans*	Multi-functional cell-wall anchored adhesin. Predicted long α-helical stalk capped with a β-rich globular head with a putative carbohydrate binding trench. Binds to ECM and host surfaces - Interacts with salivary agglutinin (SAG) complex, collagen, and fibronectin.	[54]
	CdrA	*P. aeruginosa*	Cell surface adhesin. Interacts with the ECM polysaccharide Psl. Filamentous ‘periscope’ protein comprised of tandem repeats extending from the cell surface with a sugar binding tip domain. Promotes cohesion between cells within biofilms.	[55–57]
	LecB	*P. aeruginosa*	Homo-tetrameric lectin associated with the cell surface. Interacts with the ECM polysaccharide Psl. Increases retention of cells and ECM within biofilms.	[58,59]
	LapA	*P. fluorescens*	Large (∼520 kDa) cell surface adhesin. Initial attachment to abiotic surfaces, promotes biofilm formation.	[60,61]
	Bap1	*V. cholerae*	Cell surface adhesin. Interacts with the ECM polysaccharide VPS. Multi-domain protein with β-propeller domain mediating VPS binding. Cross-links cells to the ECM matrix. ‘Double-sided tape’ mechanism with binding to VPS and the external environment. Mediates adhesion to abiotic surfaces.	[62–64]
	RbmA	*V. cholerae*	Consists of tandem fibronectin type III domains with a surface groove lined with positive charges — putative substrate binding site. Interacts with the ECM polysaccharide VPS. Acts as a flexible tether linking cells and the ECM. Crucial for ordering cells into clusters within the biofilm. Maintains 3D architecture of the biofilm.	[62,65–67]
	RbmC	*V. cholerae*	Cell surface adhesin. Interacts with the ECM polysaccharide VPS. Multi-domain protein with similar domain architecture to Bap1 with additional β-prism and βγ-crystallin domains that bind to mucins. Cross-links cells to the ECM matrix. ‘Double-sided tape’ mechanism with binding to VPS and the external environment. Role in adhesion to abiotic surfaces.	[63,64,68]
	BslA	*B. subtilis*	Hydrophobin that forms a hydrophobic layer on the surface of biofilms — protects against environmental stresses. Small protein with an immuglobulin-like fold and an extremely hydrophobic cap region.	[69,70]
Polysaccharides	PIA	*S. aureus* and *S. epidermidis*	Cationic, partially deacetylated *N*-acetylglucosamine homopolymer. Major component of the ECM, producing a fibrous net capturing cells and building up biofilm mass.	[70,71]
	Psl	*P. aeruginosa*	Mannose and galactose-rich neutral polysaccharide. Binds to cell surface attached adhesins CdrA/LecB. Implicated in initial attachment, cohesion between cells and microcolony formation.	[55,57,58,72,73]
	Pel	*P. aeruginosa*	Cationic polysaccharide comprised of galactosamine and *N*-acetyl-galactosamine. Mediates cell-cell contacts, adherence to surfaces and protection against antibiotics. Interacts with eDNA at sites of adhesion. Possible role in cross-linking host biopolymers and role in host colonisation.	[74–77]
	Alginate	*P. aeruginosa*	Comprised of β-d-mannuronic acid and α-l-guluronic acid. Replaces Psl and Pel in mucoid strains.	[72,78]
	EPS	*B. subtilis*	Major and essential component of biofilm ECM. Deletion greatly reduces biofilm size. Interacts with eDNA — responsible for 3D architecture of biofilm.	[79–81]
	VPS	*V. cholerae*	Major ECM polysaccharide comprised of glucose, galactose, *N*-acetylglucosamine and mannose. Interact with adhesins RmbA, RbmC and Bap1. Required for biofilm development and maintenance of 3D architecture.	[62,64,82]
	Cellulose	Many species	Major ECM polysaccharide comprised of β-1,4-linked d-Glucose. Cellulose fibres can bundle into ribbons that scaffold biofilm growth and 3D architecture. Can be decorated with phosphoethanolamine, allowing cellulose to form nanocomposites with amyloid fibres.	[36,83]
Nucleic acids	eDNA	All species	Ubiquitous in all biofilm ECMs. Crucial for biofilm stability. Cross-stranded structure resembling Holliday junctions Transitions between low-energy B-form to high-energy Z-form during biofilm maturation. Triggers amyloid-like fibre formation of PSM by acting as a nucleator of subunit polymerisation in *S. aureus*. Implicated in binding to curli fibres and Pel polysaccharide.	[50,77,84–89]

This list includes the proposed function for each respective molecule and is not exhaustive.

### Proteinaceous components of the biofilm matrix

#### Functional amyloids in Gram-negative bacteria

A major component of many biofilms across the realms of life are proteinaceous fibres. Commonly, such fibres are formed from relatively small protein subunits secreted into the ECM, which then interact with each other to form larger-scale assemblies. Among the most well-known fibres in biofilms are so-called bacterial functional amyloids [13,17]. Functional bacterial amyloids were first discovered in *Escherichia coli* and were termed *curli*, after their winding appearance in electron micrographs [90]. Curli fibres make up a significant portion of the biofilm dry weight, being the major polymeric component of the biofilm matrix of *E. coli* and *Salmonella* spp*.*, together with the polysaccharide cellulose [91]. Curli fibres appear to be widespread among Gram-negative bacteria [92,93].

**Figure 1.**
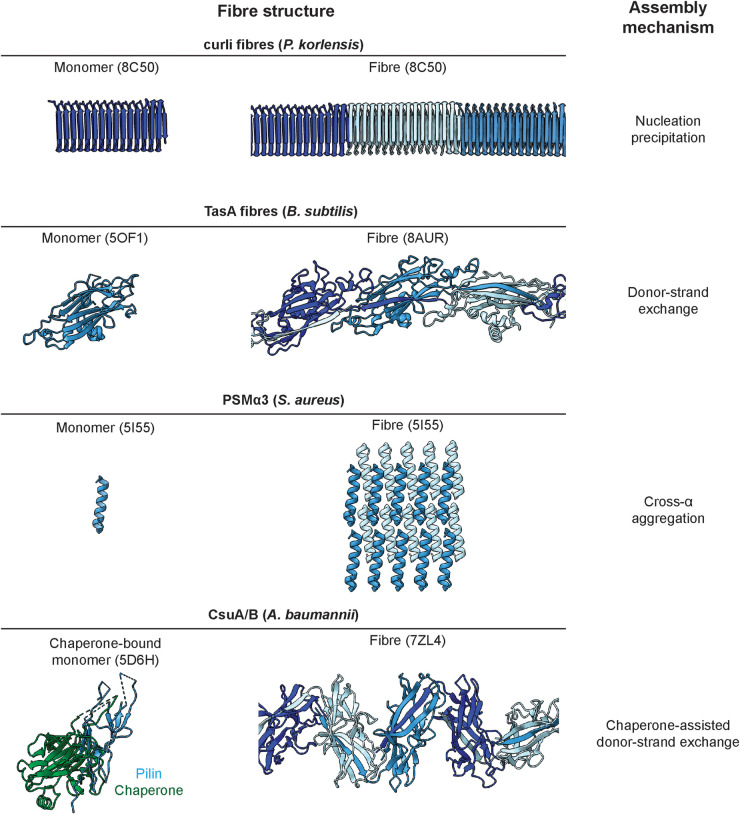
Structural features of biofilm matrix fibres. Monomeric and fibre forms are shown for each example: curli fibres composed of CsgA subunits (structural model from AlphaFold2 and cryo-EM [92], PDB ID 8C50), TasA (monomer PDB ID 5OF1 [97], fibre PDB ID 8AUR [24]), putative PSMα3 fibres as predicted from its crystal structure [20] (PDB ID 5I55), and the Csu pilus (monomer PDB ID 5D6H [30], fibre PDB ID 7ZL4 [31]).

As the name ‘functional amyloid’ suggests, curli fibres have a significant resemblance to human amyloids, as they also exhibit a so-called cross-β structure, where the filament is formed through β-sheet interactions between subunits stacked along the fibre axis [94]. Initially, the amyloid properties of curli fibres were puzzling as amyloid assembly was often considered non-functional and accidental in nature [13]. However, later studies found that many bacteria and eukaryotes have systems dedicated to the assembly of amyloids [95]. As a result of the β-sheet-rich architecture of amyloids, which produces exceptionally strong subunit interactions, amyloids have remarkable biophysical properties, including exceptional stability and resistance to protease treatment and denaturing agents [95,96]. In human amyloids, amyloidogenic subunits typically form a flat arrangement contributing one layer of β-strands to the amyloid fibre; however, how subunits arrange into an amyloid fold in bacteria had long been an open question. Recent structural work on curli fibres, combining electron cryomicroscopy (cryo-EM) and AlphaFold modelling, shows that the main amyloid-forming subunit, CsgA, forms an amyloid structure by assembling into a multi-turn β-helix which, upon polymerisation of the fibre, is continuously elongated by the addition of more subunits [92] (Figure 1). The mature CsgA monomer in *E. coli* contains five repeat regions that are highly similar to each other which results in a rise of ∼23.75 Å per subunit within the amyloid fibre, in contrast with a rise of ∼4.75 Å typically seen in human amyloids [94]. Other subunits contain variable numbers of repeats; for example, the CsgA subunit in *Pontibacter korlensis* has 15 full CsgA repeats (Figure 1). The hydrophobic surface of the fibres allows them to interact laterally to form enormous bundles, leading to a vast interacting network of fibres within the biofilm matrix.

Prior to assembly, the individual amyloidogenic subunit CsgA is disordered, requiring dedicated molecular machinery for controlled secretion and assembly in the biofilm matrix. Hence, *csgA* is encoded with several assembly factors in two operons (*csgBAC* and *csgDEFG*) [98], including factors that nucleate fibre assembly outside of the cell (CsgB) [99], prevent nucleation inside the cell (CsgC) [100], stabilise the unassembled subunits (CsgE) [101], form a translocation pore in the outer membrane (CsgG) [102], anchor fibres to the cell membrane (CsgF) [103,104], as well as positively regulate transcription of the fibre-forming subunits (CsgD) [105]. Curli fibres are extremely stable, resisting disassembly even with strong denaturing agents such as sodium dodecyl sulfate [13]. However, they are smaller and more flexible than many human amyloids characterised in neurodegenerative disease [92,106].

Since the initial discovery of curli, many functional bacterial amyloids have been characterised [107]. For example, biofilm fibres with similar amyloid properties were found in the Gram-negative Pseudomonads, where they were accordingly named *Functional amyloid in Pseudomonas* (Fap) [17,18]. Similar to curli, *fap* operons encode a main fibre-forming amyloidogenic subunit (FapC) [17], a potential nucleator or sub-stoichiometric fibre component (FapB) [17,18], a chaperone (FapA) [108], a protease (FapD) [109], a secretion pore (FapF) [109] and an accessory protein and minor fibre component that is also exported through the pore (FapE) [18]. Compared with CsgA, three larger repeat regions make up the amyloidogenic FapC subunit. Furthermore, Fap fibres are usually encoded in a single operon (*fapABCDEF*), a notable difference from the curli operons [17,18]. While Fap fibres were originally discovered in the environmental strain UK4 from non-pathogenic *Pseudomonas fluorescens*, they are also encoded in pathogenic species including *Pseudomonas aeruginosa*. Since biofilms formed by *P. aeruginosa* are a major health threat [110], compounds targeting biofilm matrix fibres like curli or Fap may be an avenue for treatment of such infections. Future research will shed light on the function and spatial organisation of functional bacterial amyloids in Gram-negative bacterial biofilms.

#### Biofilm matrix fibres in Gram-positive bacteria

Many Gram-positive bacteria scaffold their biofilms using protein fibres that share functional similarity with bacterial amyloids discussed above. The biofilm matrix in the Gram-positive model organism *Bacillus subtilis* consists of three main components: exopolysaccharides that promote cohesion [111], the BslA protein that forms a hydrophobic layer on the surface of the biofilm [112,113], and TasA matrix fibres that scaffold the biofilm [23,114]. TasA fibres consist of a 26 kDa monomeric subunit that is encoded together with a minor fibre component, TapA, and a putative signal peptidase (SipW) [115]. TasA fibres were initially thought to be amyloid-like, and hence structurally similar to curli and Fap fibres expressed by Gram-negative bacteria [23]. Supporting this hypothesis, TasA fibres exhibit a range of characteristics consistent with amyloid character, including high β-sheet content, protease resistance, and affinity to amyloid-specific antibodies and dyes [23]. Later studies provided evidence contrasting this assessment, suggesting fibres instead consist of globular subunits positioned in a head-to-tail arrangement [116]. Recent cryo-EM studies revealed that TasA fibres indeed are not amyloid-like, but are donor strand-exchanged, meaning each globular β-sandwich domain subunit extends an N-terminal strand into the next subunit to complete its fold [24] (Figure 1). This fibre arrangement is reminiscent of Gram-negative Chaperone-Usher Pathway (CUP) pili [117], although TasA fibres appear to have evolved independently [24]. TasA fibres assemble from soluble TasA subunits secreted into the biofilm matrix [23,97], requiring only the sub-stoichiometric action of TapA, a minor component that provides the first donor strand to trigger fibre assembly [118]. Once assembled within the biofilm matrix, TasA fibres then congregate into larger bundles [24,119,120]. This process is spontaneous and does not appear to depend on strong chemical interaction [24], although future studies will be required to determine further details of the underlying mechanisms at play. Once assembled, TasA bundles provide stability to the biofilm and are crucial for maintaining the delicate, crinkly three-dimensional architecture that is typical of *B. subtilis* biofilms [23]. While TasA fibres have predominantly been characterised in *Bacillus* species, TasA-like systems were found to be phylogenetically wide-spread, existing even in archaea living in extreme environments [121], underlining the ubiquity of this donor-strand exchange system in promoting fibre and biofilm stability. A study on an archaeal TasA-like system in the hyperthermophilic archaeon *Pyrobaculum calidifontis* suggests that biofilm formation is promoted by the formation of antiparallel bundles, with fibres extending from the surface of one cell to interact with fibres extending from other cells, thus physically tethering cells together [121]. It is plausible that this scenario may also be at play in *B. subtilis*, although neither the directionality of bundle interactions nor potential molecular interactions with the cell wall of *B. subtilis* have thus far been resolved [24].

#### Phenol-soluble modulins in Gram-positive bacteria

Phenol-soluble modulins (PSMs) are important virulence factors in the human pathogen *Staphylococcus aureus* [122,123]. Compared with the larger protein subunits forming the previously described functional amyloid-like fibres in Gram-negative bacteria, PSMs are small peptides with a distinct propensity to self-assemble into higher-order fibres. Three major operons of PSMs have been described: psmα encoding four peptides (PSMα1–4) that are each ∼20–25 amino acids in length; psmβ encoding PSMβ1–2, which are ∼44 residues in length, as well as δ-toxin, which is ∼22 residues in length and encoded within the RNA effector molecule of a quorum sensing system [124]. Individual, monomeric PSM peptides have been described to be biologically active without polymerising into higher-order structures, inducing host cell lysis and modulating host immune responses [122]. However, the most remarkable property of PSM peptides is their propensity to assemble into fibres with amyloid-like properties that scaffold biofilm formation [19]. The first described X-ray structure of such a fibre reconstituted *in vitro* showed an intriguing departure from typical structural motifs of amyloids containing a cross-β structure: Despite the fibres staining positive for Thioflavin T, a conventional marker for cross-β amyloids, high-resolution structures of PSMs revealed that they actually contain α-helical peptides that stack laterally to form a filament, resulting in a ‘cross-α’ fold [20] that had not previously been seen in naturally occurring peptides (Figure 1). Through later cryo-EM structure determination, the cross-α structure was confirmed for both PSMα3 and PSMβ2, with individual filaments shown to self-assemble into polymorphic tube-shaped structures [21]. Oddly, other PSMα peptides (PSMα1 and PSMα4) were found to assemble into canonical cross-β amyloid fibres and sequence alterations could be used to modulate cytotoxic properties and fibre-forming propensities [125].

Many different amyloidogenic subunits exist in *S. aureus*, polymerising into a variety of fibres. While the reason behind harbouring such an arsenal of fibre-forming subunits is not fully clear, one study has shown that expressing a variety of different PSMs results in cross-talk between them and accelerates the assembly of the peptides into fibres, improving the efficiency of biofilm formation [22]. The factors that influence the assembly and structure of fibres formed by PSMs are not fully elucidated and remain an active area of research.

#### Adhesive pili in the biofilm matrix of Gram-negative bacteria

Adhesive pili play important roles in biofilm formation. A primary function of pili is to anchor cells to a surface or other substrates, a crucial step in the early stages of biofilm formation. The role of type IV pili in this step, including in twitching motility and surface adhesion, is well-described in literature; however, their function is thought to be limited to early stages of biofilm formation and is therefore not discussed here, but is discussed in several authoritative reviews [3,34,35]. The so-called CUP pili, on the other hand, have a crucial role in stabilising mature biofilms of Gram-negative bacteria. In *P. aeruginosa*, up to five different CUP pili exist that can be classified into two types: one type, termed classical CUP pili [126], have roles in initial adhesion and the formation of microcolonies (CupA-D) [25–27], while the second type, termed ‘archaic CUP pilus’, is prevalent in mature biofilms (CupE), where it stabilises the three-dimensional architecture of the biofilm [32]. Archaic CUP pili are widely expressed and are often major components of the ECM, reported for example in *Acinetobacter baumannii*, where the archaic Csu pilus is a major virulence factor [28]. Interestingly, in addition to maintaining biofilm architecture, the archaic Csu pilus also appears to help bacteria persist on plastic surfaces in hospital environments, and thus aid its spread as a hospital-acquired pathogen [29].

All CUP pili are assembled from small (12–20 kDa) subunits through a donor-strand exchange mechanism [117,127], similar to TasA fibres discussed above. Prior to assembly, each pilin subunit consists of an incomplete Ig-like β-sandwich fold that lacks its final β-strand, but contains an extra N-terminal β-strand [127]. Upon assembly, this additional strand (designated as the donor strand) inserts into the subsequent subunit, completing its fold [127]. Classical CUP pili form tubular structures, where a single subunit assembles into a helical, hollow, rod-like architecture [128,129]. The archaic type of CUP pili, however, significantly differs in their architecture: While the subunits themselves are similar to those of the classical type [30], archaic CUP pili consist of a linear, zigzag-arrangement of pilin subunits (Figure 1) capped by a hydrophobic adhesive subunit at the pilus tip [29,31]. This adhesive tip likely binds to components of the biofilm ECM, however, the identity of substrates for archaic CUP pili is currently unknown. Some archaic CUP pili were shown to display remarkable elastic properties in optical tweezer experiments, where the archaic CUP pilus Csu from *A. baumannii* could extend up to twice its length [31]. This was made possible by breaking some of its subunit interactions, essentially acting as an elastic molecular rubber band [31]. Once the applied force ceased, the pilus coiled back up into a zigzag shape by re-forming subunit interactions [31]. A similar zigzag pilus architecture was reported for the archaic CUP pilus CupE from *P. aeruginosa* [33], a phylogenetically related bacterium, suggesting pilus elasticity might be a conserved feature. It remains to be seen how exactly archaic CUP pili help maintaining biofilm architecture, since molecular details of pilus arrangement and binding in biofilms is not yet described. The indiscriminate hydrophobic nature of the adhesin [29] means a variety of hydrophobic biofilm components or cell surfaces could be substrates, resulting in a cross-linking effect to aid cohesion of the biofilm matrix. Since the biofilm matrix is not easily amenable to conventional interaction assays, such as pull-downs or cross-linking mass spectrometry, *in situ* molecular imaging techniques such as electron cryotomography (cryo-ET) may prove to be particularly useful in determining such interactions within the biofilm ECM [130].

#### Bacteriophage liquid crystalline droplets in biofilms

Classically, bacteriophages are considered to be detrimental to bacterial cell survival. While many bacteriophages indeed kill their host during their replication cycles, some phages are continuously secreted from the host membrane, without lysing the host, allowing both to coexist [131]. Going one step further, akin to endogenous retroviruses in the human genome [132], some bacteriophages have even integrated into the host genome (called prophages), forming a symbiotic relationship with their bacterial host and providing it with survival advantages in biofilms [44,133]. A prominent example of this is Pf4, a prophage encoded in the *P. aeruginosa* genome, which enhances *P. aeruginosa* survival in harsh environments [133]. Pf4 is a filamentous, rod-shaped bacteriophage of the *Inoviridae* family [134], and as a prophage, its expression is tightly controlled by *P. aeruginosa* cells. Upon switching from planktonic to biofilm lifecycle, genes encoding Pf4 bacteriophage are massively up-regulated [135], secreting thousands of phage copies into the extracellular biofilm matrix. Intriguingly, the presence of Pf4 was shown to increase the pathogenicity of *P. aeruginosa*, correlating with increased morbidity in patients, and is prevalent in clinical isolates [45,136]. Upon host infection by *P. aeruginosa*, the presence of large amounts of phage subverts the host immune system into triggering an anti-viral rather than antibacterial immune response, increasing *P. aeruginosa* virulence [137]. Pf4 phage also promote biofilm stability by self-assembling into higher-order structures within the biofilm matrix [44,46]. In the presence of polysaccharides that are naturally present in the biofilm matrix such as alginate, Pf4 spontaneously phase-separates into spindle-shaped liquid crystalline droplets termed tactoids [44,46]. These Pf4 droplets are birefringent and individual phage filaments show mobility within the droplet, both characteristic of a nematic liquid crystalline phase [44,46]. Light microscopy and cryo-ET revealed that Pf4 liquid crystalline droplets encapsulate bacterial cells, thus physically shielding them from antibiotics [46]. The formation of Pf4 liquid crystalline droplets does not occur through chemical interactions between phage [47], which have negatively charged surfaces. Instead, phage interact with each other through depletion attraction (discussed later), an entropically driven attractive force that arises between large particles in the presence of depletants, such as other biopolymers in the biofilm matrix, which are excluded from the volume in the process [138]. Due to the abundance of Pf4 in the ECM, Pf4 liquid crystalline droplets have even been proposed to organise the entire biofilm into a liquid crystalline structure [48], contributing to biofilm stability and ability to withstand desiccation [139]. An emergent property bestowed by Pf4 liquid crystalline droplets is antibiotic tolerance of *P. aeruginosa*. Bacterial cells can be encapsulated by Pf4 droplets, resulting in protection by the formation of a barrier preventing antibiotic from reaching the cell [46,47]. The formation of liquid crystalline droplets and their interactions with bacteria depend on biophysical properties of the liquid crystalline droplet, determined by phage shape and packing, rather than specific biochemical interactions between the *P. aeruginosa* cell surface and phage capsid [47].

Inoviral prophages are pervasive across prokaryotic biomes [140], and such prophages increase bacterial pathogenicity in other Gram-negative bacteria. Examples include MDAΦ in *Neisseria meningiditis*, which promotes colonisation of epithelial cells and invasion of the blood-brain barrier, and CTXΦ in *Vibrio cholerae*, which increases bacterial virulence, amongst others [133,141]. In addition to Pf4, *P. aeruginosa* also encodes several other prophages [142]. Although the role and mechanism of action of these prophages remain underexplored, it is possible that given their biophysical similarities, they act in an analogous manner to Pf4 as a structural and protective component in biofilms. The fact that filaments can interact with the bacterial cell in a protective manner may provide a rational for why filaments are preferred components of the biofilm matrix and formed by most biofilm-forming bacteria.

### Polysaccharide organisation in the biofilm ECM

Extracellular polysaccharides (exopolysaccharides) are a key component of the biofilm ECM and have been implicated in cell-cell cohesion and intra-matrix interactions [55,143–145]. *P. aeruginosa* is known to express at least three major exopolysaccharides, namely Psl, Pel and alginate, in a strain-dependent manner, with all having important roles in biofilm development [72,146]. Psl, a mannose- and galactose-rich neutral exopolysaccharide, is involved in initial surface attachment, cell-cell attachment and microcolony formation [73]. Using Psl-specific labelled lectins, Psl was shown to associate with bacterial cells with a distinctive helical pattern upon cell adhesion to surfaces [73]. During biofilm maturation, Psl localises to the periphery of microcolonies, resulting in a Psl-free cavity at the centre of the biofilm, which may act as a pool of free cells for dispersal from the biofilm [73]. Psl has been implicated in promoting cell-cell cohesion within the biofilm [147–149], and interaction with the cell surface adhesin CdrA is essential to this phenotype [56]. CdrA is a monomeric cell surface ‘periscope’ protein forming a filamentous arrangement comprised of tandem repeat domains extending from the cell surface with a tip domain that interacts with Psl [150]. Thus, CdrA cross-links the cell with the matrix Psl, resulting in cohesion between cells [55–57]. Nanobodies targeting this interaction by blocking the adhesive domain of CdrA disrupt the biofilm and render it more susceptible to antibiotic treatment [55]. Equally, a monoclonal antibody targeting Psl was found effective in mouse models [151] and employed in clinical trials [152], underlining not only the importance of the CdrA:Psl interaction [151], but also the importance of polysaccharides in the ECM in maintaining biofilms. Apart from CdrA, a different cell surface-tethered lectin LecB has also been implicated in interaction with Psl to stabilise the biofilm matrix [58], presumably also by cross-linking Psl with the cell-surface [58,153].

Pel is a major cationic exopolysaccharide in *P. aeruginosa* biofilms that consists of galactosamine and *N*-acetyl-galactosamine moieties [74]. Pel mediates cell-cell contacts, adherence to cell surfaces and protection against aminoglycoside antibiotics [75]. It localises to the periphery of biofilms in the same manner as Psl, but is also seen at sites of attachment to surfaces [76]. Due to its net positive charge, Pel can cross-link eDNA at the site of surface adhesion in biofilms through electrostatic interactions, suggesting that Pel/eDNA interactions could be an important structural feature in early biofilm development. Interestingly, Pel could also cross-link polymers such as mucins and hyaluronan, which are secreted by the host during infection, suggesting that exopolysaccharides could play specific roles in enabling host colonisation [77].

A third type of polysaccharide, alginate, is well-characterised for its role in *P. aeruginosa* infection of hosts. Upon entering the lung environment, biofilms often adopt a mucoid, alginate-rich phenotype resulting from a mutation in the *mucA* gene leading to overexpression of this polymer [78]. Alginate consists of β-d-mannuronic acid and α-l-guluronic acid moieties, and alginate typically replaces Psl and Pel in mucoid strains [72]. As alginate is highly expressed during lung infections by *P*. *aeruginosa* in cystic fibrosis patients, alginate assembly and integrity has been suggested as a possible target for biofilm therapeutics [154–156].

Exopolysaccharides also play important roles in the biofilm matrix of many other bacterial species. In *B. subtilis*, the exopolysaccharide EPS is a major component of the biofilm matrix, and is essential for biofilm formation under most conditions [79]. Deletion of EPS results in a greatly reduced biofilm size, and deletion of both EPS and TasA results in a complete ablation of biofilm formation [80]. *B. subtilis* EPS interacts with eDNA in the biofilm matrix and this interaction is required for the complex three-dimensional structure and spatial heterogeneity of the biofilm [81]. The interaction of EPS with eDNA predominantly occurs in the initial stages of biofilm development, although EPS alone prevails in mature biofilms [81].

Another model organism for biofilm formation is *V. cholerae*, where the ECM is particularly enriched in polysaccharides when compared with other bacteria [82]. In *V. cholerae* biofilms, the exopolysaccharide VPS (Vibrio polysaccharide) is estimated to comprise up to 50% of the biofilm matrix mass [82]. VPS is secreted from bacteria after initial attachment of cells and VPS expression is maintained during biofilm maturation [62]. VPS is required for all stages of biofilm formation, including development and maintenance of the three-dimensional architecture of *V. cholerae* biofilms. VPS also plays a role in effective colonisation and infection in disease models [82,157,158]. Similar to the Pseudomonads, Vibrio also employs adhesins that directly connect cells with exopolysaccharides. Interestingly, two adhesins — Bap1 (Biofilm-associated protein 1) and its homologue RbmC (Rugosity and biofilm modulator C) directly bind to VPS [63], thus resulting in a cross-linking of cells with the matrix similar to CdrA in *P. aeruginosa*. Indeed, in super-resolution imaging of *Vibrio* biofilms, these two adhesins were found to co-localise with VPS [62]. Bap1 is a multi-domain protein consisting of a β-propeller that mediates binding to VPS and a β-prism domain that binds to N-glycans [64]. RbmC contains the same overall domain arrangement but with an additional β-prism domain as well as a βγ-crystallin domain that is mucin-binding [64]. Recent literature suggests that both proteins can be considered ‘double-sided tape’, meaning they can interact with both VPS and the outside environment [64]. The functional difference between the two adhesins lies in the type of environment they interact with: RbmC was found to preferentially cause adhesion to biotic surfaces through its mucin-and N-glycan-binding domains, while Bap1 preferentially causes adhesion to abiotic surfaces and lipids through a loop within its β-propeller domain, and shows preferential expression near the biofilm-surface interface [64]. A third protein component of the ECM in *V. cholerae*, RbmA, is crucial for ordering cells into clusters within the biofilm [62,65,66]. RbmA consists of tandem fibronectin type III domains that can directly bind to VPS and, through a previously described structural switch [66], assemble into multimers that influence the biofilm architecture of *Vibrio* through an unknown mechanism.

Taken together, these studies from different bacteria have shown that polysaccharides play a crucial role in the structuring of most, if not all, biofilms, and that interactions of polysaccharides with the cell surface, be it by binding to surface proteins or sorption to the cell, are essential for spatial organisation of the biofilm ECM.

### eDNA organisation in the biofilm ECM

Alongside proteinaceous material and polysaccharides, eDNA is another major component of the biofilm ECM [84]. eDNA is ubiquitous in the biofilm ECM from all bacterial species, and is critical for biofilm ECM stability [85]. Within the biofilm ECM, eDNA forms a cross-stranded structure stabilised by the DNABII family of proteins, which structurally resemble Holliday junctions [86,159]. Degradation of eDNA by nucleases disrupts initial biofilm formation, but does not affect mature biofilms [160,161]. A recent study provided an answer as to why this is the case: Biofilm eDNA was shown to transition from B-form to Z-form DNA during biofilm maturation [87], going from a right-handed low-energy conformation that is nuclease-sensitive to a left-handed high-energy conformation that is resistant to nucleases [162,163]. Indeed, the equilibrium between Z-form and B-form plays an important role in determining the rigidity of the biofilm [87].

eDNA also plays an important role in triggering amyloid-like fibre formation in the biofilm ECM by acting as a nucleator of subunit polymerisation, decreasing the concentration at which phase separation and fibre formation of amyloid-like proteins occur [88]. Direct interaction of eDNA with *Salmonella* curli fibre has been observed, which protects the eDNA from nuclease degradation [89]. Hence, eDNA and amyloid fibres show a mutualistic relationship in the biofilm ECM, with eDNA promoting fibre formation and phase separation, which in turn protects the eDNA from degradation.

eDNA in the biofilm ECM has been implicated in antibiotic tolerance with the highly negatively charged DNA proposed to sequester positively charged antibiotics such as aminoglycosides [44,48]. eDNA in the ECM can provide the ideal environment for liquid crystalline droplets formation, as such droplets form in the presence of high molecular mass DNA [44,48]. The multiple roles of eDNA in phase separation, amyloid-like fibre formation and sequestration of molecules shows the crucial importance of eDNA in the biofilm ECM.

### Mechanisms of biofilm formation revealed by multi-scale studies of EPS molecules

**Figure 2.**
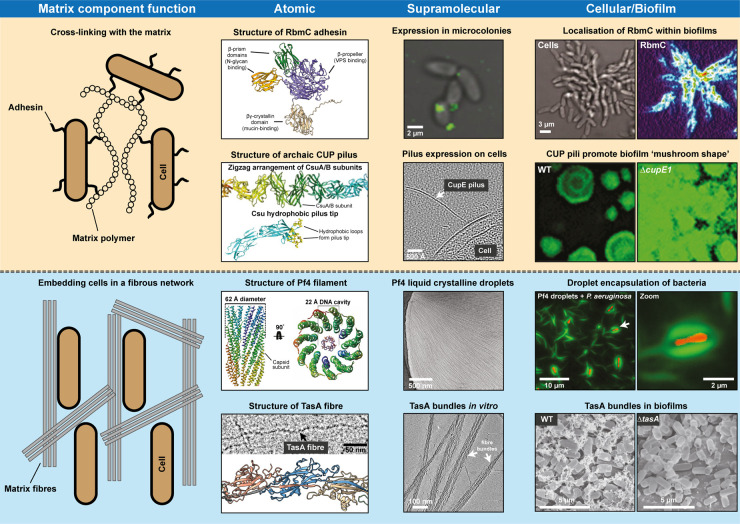
Multi-scale studies elucidate the interactions of cells with the extracellular matrix that give rise to the complex three-dimensional architecture found in biofilms. First column (from left): General principles of cell-matrix interactions: Many of the fibrous components in biofilms either cross-link cells with matrix polysaccharides or DNA (upper) or form bundles in which cells are embedded (lower). A mixture of chemical and entropic effects may contribute to both mechanisms. Second column: Atomic models of biofilm fibres produced in recent years, including the *V. cholerae* RbmC adhesin AlphaFold prediction [169,170] annotated by domain function as described previously [64], an archaic CUP pilus from *A. baumannii* [29,31], Pf4 phage [46], and TasA fibres [24]. Third column: Localisation of RbmC expression in *V. cholerae* microcolonies as shown by fluorescence microscopy (from reference [62]; reprinted with permission from AAAS), CupE pilus extending from a *P. aeruginosa* cell visualised by electron cryotomography [33], and formation of bundles by Pf4 [46] and TasA [24]. Fourth column: RbmC expression in *V. cholerae* biofilms (from reference [62]); reprinted with permission from AAAS), fluorescent *P. aeruginosa* biofilms showcasing a distinct mushroom-shaped 3D architecture that depends on the expression of CupE pili (reprinted with permission from reference [32]), encapsulation of *P. aeruginosa* cells by Pf4 liquid crystalline droplets [46], and SEM imaging of *B. subtilis* biofilms with and without *tasA* [120].

Bacterial cells within biofilms show significant levels of spatio-temporal organisation and patterning [164]. Bacterial biofilm architecture results from a complex interplay of physical, chemical, and biological mechanisms, with the ECM playing a central role [165–168]. We propose that there are two organisational principles that could explain cohesion of bacterial cells in biofilms (Figure 2). The first mechanism, termed bridging, is based on cross-linking or sorption of cells with polysaccharides in the ECM, tethering cells together and causing their aggregation. The second mechanism is based on depletion attraction, causing the formation of tightly packed structures in the presence of biopolymer [164,171–173].

Bridging occurs when a polymer interacts with multiple bacterial cells, causing the cells to be spontaneously pulled together [171,174]. Bridging can involve more than one chemical component, and may for example be mediated by a proteinaceous adhesin binding to a polysaccharide, as seen in the CdrA/Psl system of *P. aeruginosa* [55,56] and RbmC/VPS system of *V. cholerae* [63].

Depletion attraction is an entropic principle that causes alignment of objects of similar shape in the presence of high levels of non-adsorbing polymers. Spontaneous movement causes bacterial cells to come closer together, as this will exclude the polymer, allowing higher degrees of freedom for biopolymer movement and increasing the entropy of the system [44,164]. Depletion attraction can induce interactions between objects of similar shapes, including cells, but also between fibrous matrix components, and between cells and fibrous matrix components. For example, depletion attraction is the driving force of liquid crystalline droplet formation by Pf4 phages in the *P. aeruginosa* biofilm matrix, where phages spontaneously align in the presence of polysaccharide polymer, which is excluded from the droplet in the process [47]. This process could also contribute to the formation of higher-order assemblies by other biofilm fibres, such as TasA and curli. Given the abundance of fibre bundles and polysaccharides in many bacterial systems, it is plausible that entropic forces such as depletion attraction play a significant role in producing cohesion and order in many other biofilms, most of which contain polysaccharides that could act as a depletant.

Previous studies have shown that the environment can have significant effects in producing order within the biofilm [5,175–179]. While it is not completely understood how they drive such changes, these environmental factors can substantially modulate the three-dimensional shape and properties of biofilms. For example, in *V. cholerae* biofilms embedded in hydrogels, but not on flat surfaces, cells align into bipolar structures reminiscent of nematic phase liquid crystalline droplets [179]. This cell ordering was proposed to occur due to biomechanical stress transmission across the biofilm mediated by ECM biopolymers such as VPS [179]. At the same time, it was shown in *V. cholerae* biofilms that mechanical interactions between cells and matrix components are of significant importance in imparting order in the biofilm [180]. Generally, a combination of physical (entropy-driven) and chemical (adhesion) effects are most likely responsible for maintaining the three-dimensional organisation of the biofilm, and it will take focused further research to accurately assess the contributions of such factors in shaping bacterial biofilms.

## Conclusion

The three-dimensional architecture of biofilms arises from complex interactions of ECM components and bacterial cells. This matrix has long been imperfectly understood, because on one hand, the structure and spatial organisation of many of the components was poorly characterised, and on the other hand, the interactions between components were unclear. Many fibre components of the biofilm matrix have now been structurally characterised and first principles of the molecular organisation of the biofilm matrix are being revealed. Recent studies have led to the realisation that a complex interplay of chemical interactions and physical principles gives rise to the exceptional emergent properties of the biofilms.

Bacterial biofilms are a major cause of human disease, accounting for 65–80% of all chronic infections [181,182]. Biofilms are notoriously difficult to treat as the ECM provides an effective barrier to many antimicrobials, making them up to 1000-fold more tolerant to antibiotic treatment than planktonic bacteria [183], leading to poor bacterial eradication and chronic infection [76]. Employing delivery mechanisms that circumvent the ability of the biofilm matrix to reduce antibiotic uptake should allow greater penetration of antimicrobials into biofilms. For this goal, further insights into the interactions between the matrix components and the physicochemical microenvironments arising through them are urgently needed. This fundamental knowledge will help improve our understanding of how bacteria protect themselves against antibiotics, aiding our understanding of multicellular bacterial life and the development of better treatments for bacterial infections.

## Open Access

Open access for this article was enabled by the participation of MRC Laboratory of Molecular Biology in an all-inclusive *Read & Publish* agreement with Portland Press and the Biochemical Society under a transformative agreement with JISC.

## Abbreviations

cryo-EM: electron cryomicroscopy
cryo-ET: electron cryotomography
CUP: Chaperone-Usher Pathway
ECM: extracellular matrix
eDNA: extracellular DNA
EPS: extracellular polymeric substances
PSM: Phenol-soluble modulin
VPS: Vibrio polysaccharide
